# Effects of Electrospinning on the Viability of Ten Species of Lactic Acid Bacteria in Poly(Ethylene Oxide) Nanofibers

**DOI:** 10.3390/pharmaceutics11090483

**Published:** 2019-09-18

**Authors:** Špela Zupančič, Katja Škrlec, Petra Kocbek, Julijana Kristl, Aleš Berlec

**Affiliations:** 1 Faculty of Pharmacy, University of Ljubljana, Aškerčeva 7, SI-1000 Ljubljana, Slovenia; spela.zupancic@ffa.uni-lj.si (Š.Z.); petra.kocbek@ffa.uni-lj.si (P.K.); 2 Department of Biotechnology, Jožef Stefan Institute, Jamova 39, SI-1000 Ljubljana, Slovenia; katja.luzarskrlec@gmail.com

**Keywords:** nanotechnology, biotechnology, probiotics, *Lactobacillus*, *Lactococcus*, electrospinning, nanofibers, drying, local delivery, viability

## Abstract

Lactic acid bacteria can have beneficial health effects and be used for the treatment of various diseases. However, there remains the challenge of encapsulating probiotics into delivery systems with a high viability and encapsulation efficacy. The electrospinning of bacteria is a novel and little-studied method, and further investigation of its promising potential is needed. Here, the morphology, zeta potential, hydrophobicity, average cell mass, and growth characteristics of nine different species of *Lactobacillus* and one of *Lactococcus* are characterized. The electrospinning of polymer solutions containing ~10 log colony forming units (CFU)/mL lactic acid bacteria enabled the successful incorporation of all bacterial species tested, from the smallest (0.74 µm; *Lactococcus lactis*) to the largest (10.82 µm; *Lactobacillus delbrueckii* ssp. *bulgaricus*), into poly(ethylene oxide) nanofibers with an average diameter of ~100 nm. All of these lactobacilli were viable after incorporation into nanofibers, with 0 to 3 log CFU/mg loss in viability, depending on the species. Viability correlated with the hydrophobicity and extreme length of lactic acid bacteria, whereas a horizonal or vertical electrospinning set-up did not have any role. Therefore, electrospinning represents a promising method for the incorporation of lactic acid bacteria into solid delivery systems, while drying the bacterial dispersion at the same time.

## 1. Introduction

Probiotics are living microbes that have beneficial health effects when administered to a host in a sufficient quantity. They most often belong to the very diverse genus of *Lactobacillus*, which includes a large number of species with a “generally recognized as safe” or “qualified presumption of safety” status [1,2]. In 2015, 175 genomes of lactobacilli were included in a comparative taxonomic study [2]. These are non-spore-forming rods or coccobacilli that are characterized by low genomic guanine and cytosine contents, the production of lactic acid, and complex nutritional requirements. They are aero-tolerant or anaerobic, aciduric, or acidophilic [1]. *Lactobacillus* spp. are particularly important in human nutrition and are also considered to be important cell factories in biotechnology, for the production of valuable chemicals. They have also been tested in several clinical trials to evaluate their efficiency for the treatment of a wide spectrum of diseases [1,3].

*Lactobacillus* probiotics are usually administered orally for the treatment of intestinal diseases, such as acute gastroenteritis [4], necrotizing enterocolitis [5], antibiotic-associated diarrhea [6], and inflammatory bowel disease [7], among others. Moreover, these probiotics have also shown promising potential for the treatment of extra-intestinal diseases, including urinary tract infections, periodontal disease, and bacterial vaginosis [8,9]. However, for such diseases, topical administration of the lactobacilli would appear advisable, to promote their higher efficiency. 

For example, the vagina is densely colonized by microbiota (10^7^–10^8^ colony-forming units [CFU]/g vaginal fluid) [10,11]. Among the distinct ecological environments of the human body, the vaginal microbiota is the least diverse [12], where bacteria from the genus *Lactobacillus* predominate in the majority of healthy women (> 70%) [10,11]. A healthy vaginal microbiota includes from four to 12 different species [13], and the species that dominate individual microbiota include *Lactobacillus crispatus, Lactobacillus jensenii, Lactobacillus gasseri*, and *Lactobacillus iners* [11,14]. Their use for re-establishing the dominance of lactobacilli in vaginal dysbiosis (e.g., bacterial vaginosis and vaginal candidiasis) is therefore rational, and might even be the most justified among the different applications of these probiotics. 

For successful treatments, probiotics need to be incorporated into patient-friendly delivery systems with large numbers of incorporated probiotics, which need to remain stable and survive for prolonged periods during storage [8]. Liquid and semi-solid dosage forms (e.g., hydrogels [15,16]) can have shorter residence times at the local site [10] and issues regarding probiotic stability compared to solid dosage forms. For preparations of these forms, different encapsulation and drying techniques have been used, including microencapsulation, emulsification, coacervation, spray drying, and lyophilization [17,18]. The main drawbacks of these techniques are the use of organic solvents and high temperatures [17]. Electrospinning represents a promising method for the incorporation of probiotics into nanofibers, allowing drying of the bacteria and preparation of a solid delivery system in a single step [19], and thereby offering considerable advantages over techniques such as microencapsulation and lyophilization. 

Electrospinning is an established technique used to produce fibers with small diameters, in the range of several nanometers to micrometers, which are often called nanofibers. The manufacturing process is based on the drying of a thin liquid jet that is formed from a drop of polymer solution in a strong electric field [20]. The use of nanofibers has been suggested for several biomedical applications, including wound dressing, drug delivery, and tissue engineering [20]. Of these, drug delivery is the most promising, whereby its advantages include high drug loading, a high incorporation efficiency, the simultaneous delivery of diverse therapeutics, an increased surface area, a good mechanical resistance, an enhanced distribution at mucosal surfaces, ease of operation, and cost effectiveness [20,21,22]. 

To date, there have only been a few studies on the electrospinning of probiotics [23,24,25,26,27,28]. *Bifidobacterium animalis* subsp. *lactis* Bb12 has been incorporated into poly(vinyl alcohol) nanofibers [24]. Among the lactobacilli, *Lactobacillus acidophilus* was incorporated into poly(vinyl alcohol) and poly(vinyl pyrrolidone) nanofibers [25], and into agrowaste-based nanofibers [23]. *Lb. gasseri* was incorporated into poly(vinyl alcohol) nanofibers [28] and *Lb. rhamnosus* into poly(vinyl alcohol) and sodium alginate-based nanofibers [27]. Recently, we optimized the incorporation of *Lactobacillus plantarum* into poly(ethylene oxide) (PEO) nanofibers, and confirmed that their viability can be improved by the addition of lyoprotectants, such as trehalose [26].

Lactobacilli-containing nanofibers represent an innovative delivery system that would be particularly appropriate for topical administration. Due to scarce data on the influence of electrospinning on the viability of different *Lactobacillus* species, in the present study, we incorporated nine different species from the genus *Lactobacillus* and one from the genus *Lactococcus* into PEO nanofibers and assessed their viabilities following their incorporation, as a single study. To explain the higher susceptibilities of some strains to electrospinning, the morphology, zeta potential, hydrophobicity, average mass of bacterial cells, and growth characteristics of these 10 strains were also characterized.

## 2. Materials and Methods

### 2.1. Materials

Poly(ethylene oxide) (Mw, 900 kDa) and chloramphenicol were obtained from Sigma Aldrich (Steinheim, Germany). Phosphate-buffered saline (pH 7.4; osmolality: 280–315 mOsm/kg) was sourced from Gibco (Life Technologies, Carlsbad, CA, USA). De Man, Rogosa and Sharpe (MRS) and M-17 media for culturing the *Lactobacillus* spp. and *L. lactis*, respectively, were obtained from Merck (Darmstadt, Germany). Water, buffers, and growth media were sterilized by autoclaving at 2 bar and 121 °C for 20 min.

### 2.2. Bacterial Strains and Culturing Conditions

The *Lactobacillus* strains were grown at 37 °C in MRS medium without aeration. *L. lactis* MG1363 was grown at 30 °C in M-17 medium supplemented with 0.5% glucose (GM-17), without aeration (Table 1). For long-term storage, the bacterial strains were kept frozen at −80 °C in their corresponding growth medium with 20% (*v/v*) glycerol. For each experiment, fresh bacteria cultures were cultivated. Frozen cultures were first transferred onto an appropriate growth medium agar plate and incubated for 2 days at 37 °C (*Lactobacillus* spp.) or 30 °C (*L. lactis*). A single bacteria colony was picked, inoculated into 10 mL of the appropriate medium, and incubated at 37 °C or 30 °C for 24 h. Overnight cultures were diluted in fresh medium (1:100, *v/v*) and grown until a stationary growth phase was reached (as determined from the growth curves; see below). The cultures were centrifuged at 5000× *g* for 10 min (Sorvall Lynx 4000; ThermoFisher Scientific, Waltham, MA, USA). The cells were then washed twice with phosphate-buffered saline and resuspended in an appropriate volume of water.

### 2.3. Characterization of Bacterial Strains

#### 2.3.1. Determination of Cell Surface Charge of the Bacteria

The cell-surface net charge of the bacteria (as represented by the zeta potential) was determined by laser Doppler micro-electrophoresis (Zetasizer Nano ZS; Malvern Instruments, Malvern, UK). Bacteria dispersions in 0.9% (*m/v*) NaCl with a concentration of 10.3 log CFU/mL were 200-fold diluted with deionized water, put into plastic cuvettes, and covered with the Zeta Dip Cell. The measurements of zeta potential were performed at 25 °C using an He-Ne laser, with a wavelength of 633 nm and the backscatter detector at the scattering angle of 173°. The electrophoretic mobilities measured for the bacterial cells in the applied electric field were employed to automatically calculate their zeta potential using the Smoluchowski approximation of the Henry equation. At least three measurements were performed for each bacterial strain.

#### 2.3.2. Determination of Cell Surface Hydrophobicity of the Bacteria

The hydrophobicity of the bacteria was determined according to the method of Perez et al. [36], with some modifications. Cultures of the strains were harvested in the stationary phase by centrifugation at 12,000× *g* for 5 min at 4 °C, washed twice with 50 mM K_2_HPO_4_ (pH 6.5) buffer, and finally resuspended in the same buffer. The cell suspensions were adjusted to an absorbance at 560 nm (A_560_) of 0.5. Three milliliters of bacterial suspensions was added to 0.6 mL n-hexadecane, vortexed for 120 s, and left for 30 min at room temperature, to allow separation of the two phases. The aqueous phase was carefully removed and A_560_ was measured using a spectrophotometer (Lambda Bio+; Perkin Elmer, Weltham, MA, USA). The decrease in the absorbance of the aqueous phase was taken as the measure of the cell surface hydrophobicity (H%), which was calculated as in Equation (1):

H% = [(A_0_–A)/A_0_] × 100 (1)

where A_0_ and A are the absorbances before and after extraction with n-hexadecane, respectively.

#### 2.3.3. Determination of Mass of the Bacterial Cells

Bacterial dispersions in water with known numbers of cells and known dispersion volumes were frozen at −80 °C for 24 h, and then lyophilized (Beta 1-8K; Martin Christ, Osterode am Harz, Germany). The first drying phase (T_shelf_ = −5 °C; P = 0.63 mbar) was performed for 24 h, and the second drying phase (T_shelf_ = 20 °C) for 1 h. The lyophilizates obtained were weighed.

#### 2.3.4. Growth Curves of *Lactobacillus* spp. and *L. lactis*

Overnight cultures of *Lactobacillus* spp. and *L. lactis* MG1363 were diluted (1:100) in 200 µL fresh MRS or GM-17 growth medium, respectively, in 96-well microplates. The plates were sealed with sealing film and incubated in a microplate reader (Sunrise; Tecan, Salzburg, Austria) at 37 °C (or 30 °C for *L. lactis*) for 24 h. A_595_ was measured every 2 min. The plates were shaken for 10 s before each measurement. Each culture was grown in quadruplicate. The growth rates and lag phases of the growth curves were analyzed using the DMFit 3.5 software and the model of Baranyi and Roberts [37].

### 2.4. Preparation of Polymer Solutions with the Bacteria

Overnight cultures of bacteria were diluted (1:100) in 500 mL fresh medium and grown to an optical density at 600 nm (OD_600_) of 2.50 to 3.00. The cultures were centrifuged at 5000× *g* for 10 min, and washed twice with phosphate-buffered saline. To obtain 4% (*w/v*) PEO bacterial dispersions, the cells were dispersed in an appropriate volume of deionized water. PEO was added to the bacterial dispersions with 10.6 ± 0.8 log CFU/mL in deionized water, and stirred at room temperature for 4 h.

### 2.5. Rheological Characterization of Polymer Solutions with the Bacteria

Rotational and oscillatory tests of PEO solutions with the bacteria were performed using a rheometer (Physica MCR 301; Anton Paar, Graz, Austria) with a cone-plate measuring system (CP50-2; cone radius, 24.981 mm; cone angle, 2.001°) at a constant temperature of 25.0 ± 0.1 °C, as previously described [27,28]. The zero-gap was set to 0.209 mm. The shear rate during the rotational tests ranged from 1 /s to 100 /s, and the viscosity (η) was calculated as η = τ_c_/$\dot{\gamma }$, where τ_c_ is the shear stress and $\dot{\gamma }$ is the shear rate. The relative viscosity was calculated as the viscosity of the PEO solutions with bacteria, divided by the viscosity of the PEO solution, at a shear rate of 1 /s. Oscillatory tests were performed at a frequency from 0.2 /s to 100 /s, and an amplitude of 1%, which was within the linear viscoelastic region determined in prior amplitude-sweep experiments, to define the phase shift angle (*δ*). The storage (*G′*) and loss modulus (*G″*) were calculated as in Equations (2) and (3), respectively:

G′ = (τ_a_/γ_a_) × cosδ (2)

*G*″ = *(τ_a_/γ_a_)* × sinδ (3)

where *τ_a_* is the shear stress and *γ_a_* is the deformation. The damping factor was calculated as tanδ = G”/G’.

### 2.6. Preparation of Bacteria-Loaded Nanofibers by Electrospinning

The bacterial dispersions in 4% (*m/v*) PEO solutions were transferred into a 5 mL syringe fitted with a metallic needle of a 1 mm inner diameter and located horizontally on a syringe pump (model R-99E; RazelTM, Linari Engineering, Valpiana, Italy). The electrode of a high-voltage power supply (model HVG-P60-R-EU; Linari Engineering, Valpiana, Italy) was clamped onto the metallic needle, and the collector was grounded and covered with a piece of aluminum foil. The process was set to a flow rate of 0.4 mL/h, voltage of 15 kV, and nozzle-to-collector distance of 15 cm. Additionally, PEO solution with *Lb. delbrueckii* ssp. *bulgaricus* was electrospun in a vertical electrospinning set-up employing the same equipment and conditions as used for the horizontal electrospinning.

### 2.7. Characterization of Nanofibers Loaded with the Bacteria

#### 2.7.1. Morphology of the Bacterial Cells and Nanofibers

Three microliters of each bacterial dispersion was pipetted onto a metal stub and air dried, and the nanofiber mats were attached to metal stubs with double-sided conductive tape. The samples were not coated prior to the imaging under scanning electron microscopy (Supra 35 VP; Carl Zeiss, Oberkochen, Jena, Germany), which was operated at an acceleration voltage of 1 kV, with a secondary detector. The length and width of at least 30 randomly selected bacteria and the diameters of 50 randomly selected nanofibers (as parts not containing any bacteria) were measured using the ImageJ 1.51j8 software (National Institutes of Health, Bethesda, MD, USA).

#### 2.7.2. Viability of the Bacteria

The viability of the bacterial cells in the PEO solutions was determined prior to the electrospinning and after their incorporation into the nanofibers. The number of viable suspended bacteria in a known volume of bacterial dispersion was determined using the drop plate method [38]. Eight ten-fold serial dilutions of bacterial cells in PEO solutions were prepared using 50 mM phosphate buffer at pH 7.4, with each dilution in a final volume of 1 mL. Ten microliters of each dilution was pipetted onto agar plates as five replicates, and after incubation, the dilution that contained 3 to 30 colonies per single drop was counted, and replicates were averaged. These data were expressed as CFU/mL and were converted into log CFU/mL. The viability of the bacteria incorporated into the nanofibers was determined by dissolving a known mass of nanofibers in 50 mM phosphate buffer, with a pH of 7.4. Bacterial diluting and counting were performed as described above; here, the data were expressed as CFU/mg nanofibers, and were converted to log CFU/mg. The experimental bacteria loading was compared to the theoretical bacteria loading. The theoretical bacteria loading (CFU/mg) was calculated as the number of bacterial cells in the polymer solution (CFU) per dry weight of polymer and bacterial cells, in 1 mL dispersion. The dry weight of PEO was assumed to be 4 mg, while the dry weight of 1 × 10^10^ bacterial cells was determined as described in Section 2.3.3, and is shown in Table 2.

### 2.8. Statistics

The effects of hydrophobicity on the viability of the bacteria were analyzed by applying Mann–Whitney nonparametric tests (*, *p* < 0.05), using the GraphPad Prism 5.00 software. All of the data are presented as means ± standard deviation (SD).

## 3. Results

### 3.1. Physical Characteristics of the Lactobacillus spp. and L. lactis

**Figure 1:**
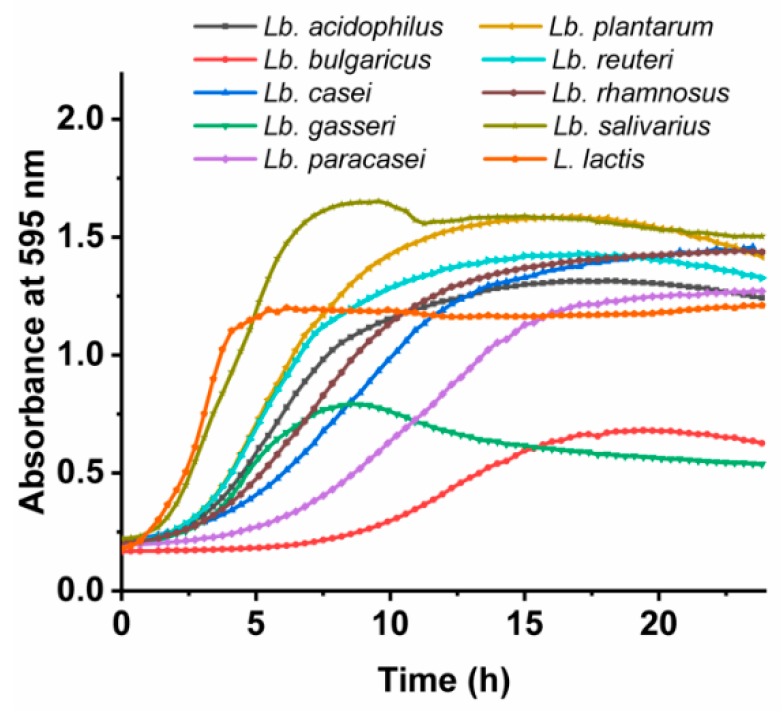
Representative growth curves of the *Lactobacillus spp*. and *L. lactis*.

The physical properties of the lactic acid bacteria (e.g., size, charge, and hydrophobicity) were hypothesized to affect their viability after incorporation into the nanofibers. Despite the wealth of information on lactic acid bacteria available, studies that compare physical properties and growth characteristics of multiple lactic acid bacteria are scarce; these were therefore determined in the present study for the selected pool of lactobacilli and *L. lactis* (Table 2, Figure 1). The lactobacilli differed little in terms of their average cell width. Two species had average cell lengths >2 µm, namely *Lb. gasseri* (4.68 µm) and *Lb. delbrueckii* ssp. *bulgaricus* (10.82 µm). The zeta potentials of the different species were comparable, at around −10 mV. The exceptions here were *Lactobacillus* *casei* and *Lactobacillus* *rhamnosus*, with zeta potentials of ~0 mV, and *Lactobacillus* *paracasei*, with the lowest zeta potential (−23.9 mV). There were considerable differences in the cell hydrophobicities, allowing division of the bacterial species into two groups: bacteria with a lower hydrophobicity (<40%; hydrophilic) and bacteria with a higher hydrophobicity (>70%; hydrophobic). Differences in mass (dry weight) of the bacteria might be attributed to the production of exopolysaccharides that differ among these different species. Exopolysaccharides can remain attached to bacteria, despite the washing step. The highest mass was determined for *Lb. rhamnosus* ATCC 53103 and *Lb. delbrueckii* ssp. *bulgaricus* ATCC 11842, which are known producers of exopolysaccharides [39,40].

### 3.2. Growth Characteristics of the Lactobacillus spp. and L. lactis

Apart from their physical properties, the bacteria also differed in their growth characteristics when cultured under the same conditions (Figure 1). *Lactobacillus salivarius* was the fastest growing *Lactobacillus* spp., with a growth rate of ~0.3 /h. *Lb. plantarum* and *Lactobacillus reuteri* had a growth rate of ~0.2 /h, while the majority of strains grew at growth rates of 0.1 /h to 0.2 /h. The slowest growers were *Lb. delbrueckii* ssp. *bulgaricus* and *Lb. casei*, with growth rates just above 0.05 /h. These last two and *Lb. paracasei* also had the longest lag times (>5 h), while the rest of the bacteria had lag times of <3 h.

### 3.3. Viscosity of Polymer Solutions with the Dispersed Bacteria

**Figure 2:**
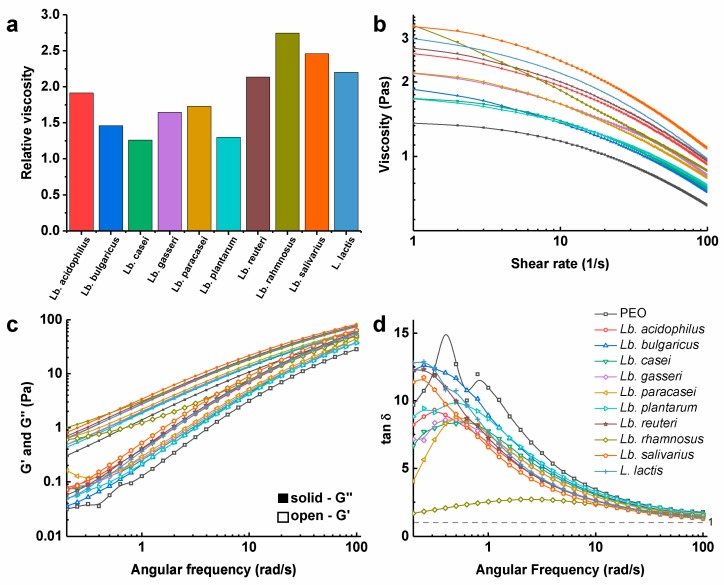
Rheological characterization of 4% (*m/v*) poly(ethylene oxide) (PEO) solutions without bacteria and with the dispersed *Lactobacillus* spp. or *L. lactis*. (**a**) Relative viscosity, as the ratio of the viscosity of the PEO dispersions with bacteria to that of the PEO solution, at a 1 /s shear rate. (**b**) Viscosity of the dispersions as a function of the shear rate. (**c**) Storage (G’) and loss (G”) moduli, and (**d**) tan δ as a function of the angular frequency.

**Figure 3:**
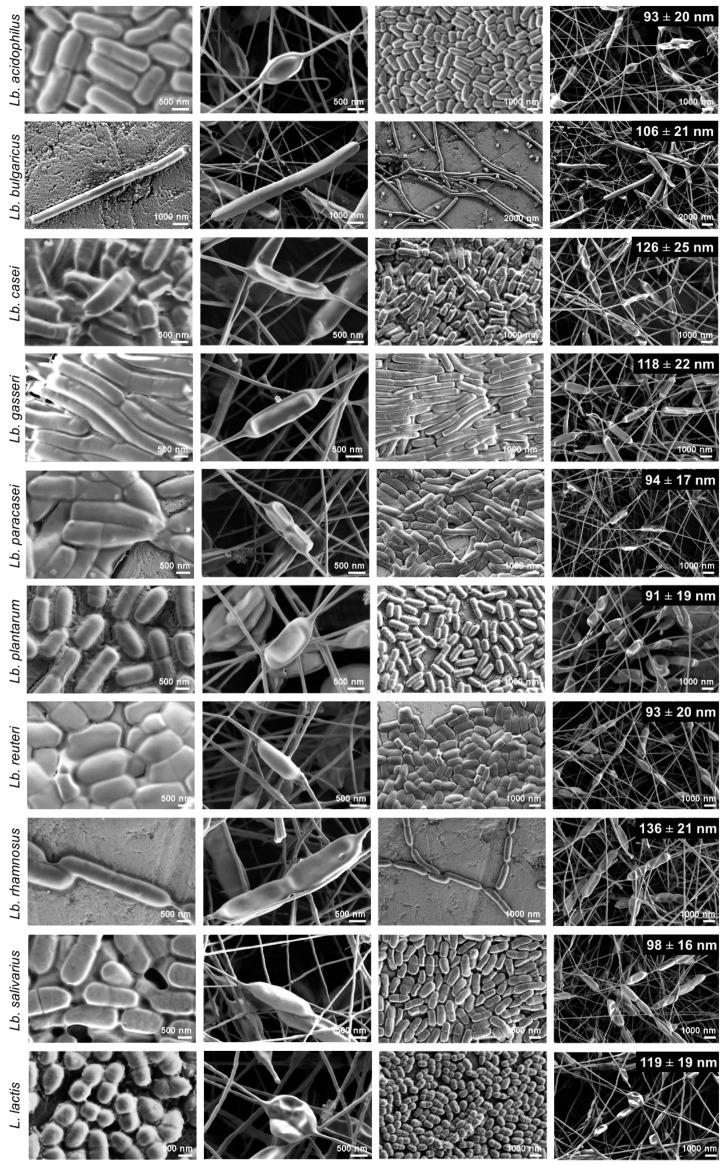
Scanning electron microscopy images of the lactic acid bacteria air dried from the water dispersion (columns 1, 3; under high and low magnification, respectively) and bacteria-loaded nanofibers (columns 2, 4; under high and low magnification, respectively). The numbers given in column 4 indicate the average nanofiber diameters.

The dispersions of the bacteria in 4% (*m/v*) PEO solution increased the viscosity in comparison to 4% (*m/v*) PEO solution without the bacteria (Figure 2). The viscosity at the low shear rate (1 /s) of the PEO solution was 1.36 Pas. PEO solutions with *Lb. rhamnosus* and *Lb. salivarius* had the highest viscosities, with 2.8-fold and 2.5-fold increases, respectively, compared to the PEO solution without these bacteria. Conversely, *Lb. casei* and *Lb. plantarum* had the lowest viscosities among these bacterial dispersions, with only a 1.3-fold increase (Figure 2a). The PEO solutions and all of the PEO solutions with dispersed bacteria showed shear-thinning behavior, which was similar for all of the samples tested, with the exception of the PEO solution with *Lb. rhamnosus*, where the viscosity decreased more rapidly through an increase in the shear rate (Figure 2b). For all of the dispersions tested, the loss modulus dominated over the storage modulus (Figure 2c), and consequently, the damping factor (tan δ), as the ratio between the loss and storage modulus was >1 at all of the tested angular frequencies (Figure 2d). Therefore, in these viscoelastic dispersions, the viscous portion prevailed over the elastic one. All of these dispersions were comparable, with the exception of the PEO solution with *Lb. rhamnosus*, where the storage modulus was a lot higher (Figure 2c) and the damping factor was lower (Figure 2d). This might be attributed to the production of exopolysaccharides and the pronounced growth of *Lb. rhamnosus* as chains, as seen in Figure 3. Viscosity did not correlate with zeta potential or bacterial hydrophobicity.

### 3.4. Morphology of Nanofibers with the Bacteria

Under scanning electron microscopy, the thin nanofibers with a thickness of ~100 nm showed local thickenings in the shape of the bacteria, which confirmed the effective incorporation of all of these bacterial species into nanofibers (Figure 3). During SEM analysis, we did not observe any bacteria, which were not incorporated into nanofibers. The nanofiber polymer coating of the bacterial cells was thin, homogenous, and showed no cracks. Single or dividing cells were oriented along the nanofibers and evenly distributed over the nanofiber mats. These mostly retained their shape, compared to the bacteria before the incorporation; however, in some cases, the flattening of cells was seen (Figure 3). The diameters of the nanofibers with the incorporated bacteria in regions without the bacteria varied slightly among these different strains (Table 3). However, these differences did not correlate with the viscosities of the bacterial/PEO dispersions.

### 3.5. Viability of the Different Lactic Acid Bacteria after Electrospinning

All of the bacterial species were viable following their incorporation into the nanofibers. For five species (*Lb. acidophilus*, *Lb. gasseri*, *Lb. reuteri*, *Lb. salivarius*, and *L. lactis*), the survival decreased by <1 log unit, indicating a high viability. Four species (*Lb. casei*, *Lb. paracasei*, *Lb. plantarum*, and *Lb. rhamnosus*) showed a decreased survival of between 1 and 2 log units. The worst survival was for *Lb. delbrueckii* ssp. *bulgaricus*, with more than a 2 log decrease in viability (Table 4).

**Figure 4:**
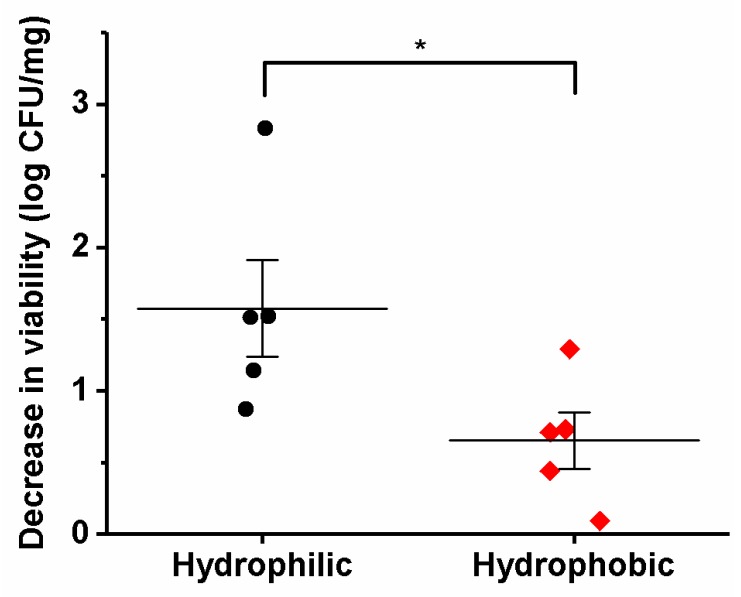
Bacterial cells with a lower hydrophobicity (< 40%, hydrophilic, black circles) showed larger decreases in viability after incorporation into nanofibers, in comparison to bacterial cells with a higher hydrophobicity (> 70%, hydrophobic, red diamonds). The horizontal lines indicate the means. *, *p* < 0.05 (Mann–Whitney tests).

Interestingly, the loss of viability correlated with the hydrophobicity of the bacterial cells. The hydrophilic bacteria showed significantly higher decreases in viability than the hydrophobic bacteria (Figure 4). This suggests that hydrophobic molecules at the bacterial surface (e.g., including exopolysaccharides) offer better protection for the bacteria during their incorporation into nanofibers. There were no correlations between the loss of viability and viscosity of dispersion or zeta potential of cells.

**Figure 5:**
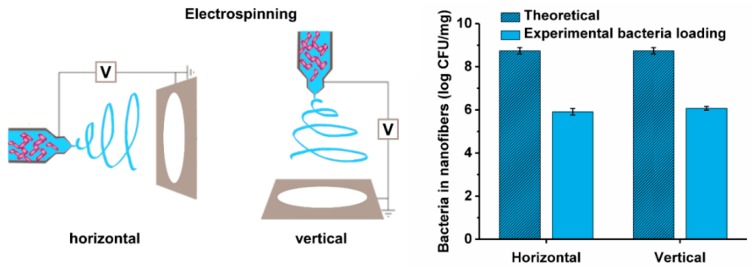
Theoretical and experimental loading of *Lb. delbrueckii* ssp. *bulgaricus* in nanofibers using either horizontal or vertical electrospinning.

In general, the width or length of the cells did not correlate with the viability of the bacteria. However, for *Lb. delbrueckii* ssp. *bulgaricus* in particular, the loss of viability might also be correlated with the bacterial cell size, as these bacteria had by far the longest cells and by far the highest decrease in viability. To exclude any influence of the direction of the electrospinning and possible losses of bacteria during the electrospinning due to the gravitational force, horizontal electrospinning was replaced with vertical electrospinning. The survivals here were not significantly different (Figure 5), which suggests that the direction of electrospinning has no major role in the bacterial viability.

## 4. Discussion

The scope of the use of probiotics can be widened by the introduction of novel delivery systems. The incorporation of probiotics into nanofibers is an emerging approach for the delivery of probiotics to body sites that require their controlled and/or sustained release. We and others have recently demonstrated the effective incorporation of lactic acid bacteria into nanofibers, and have shown that these incorporated bacteria retain their viability. However, previous studies have been limited to just five species of lactic acid bacteria: *Lb. acidophilus*, *Lb. gasseri Lb. plantarum*, *Lb. rhamnosus,* and *Bifidobacterium animalis* [19,23,24,25,26,27,28]. Different species of lactic acid bacteria can differ considerably in their properties and therapeutic effects, and therefore, it is crucial to strengthen our knowledge of the behavior of the different lactic acid bacteria during electrospinning.

We effectively incorporated nine taxonomically different species of the genus *Lactobacillus* and one species of the genus *Lactococcus* into PEO nanofibers, and have thus considerably increased our knowledge of the number of lactic acid bacteria that can be delivered using electrospun nanofibers. The dispersions of these bacteria in 4% (*m/v*) PEO solution had different rheological properties. A higher viscosity was attributed to the production of exopolysaccharides or to the pronounced chain growth phenotype. Although the bacteria in the PEO solution affected the viscosity, there was no need to change the process parameters during the electrospinning of the previously optimized electrospinning of PEO solutions without the bacteria, which enabled the production of smooth PEO nanofibers in a continuous process [26]. The previously reported mean diameter of PEO nanofibers without the incorporated bacteria was 135 ± 25 nm [26], which was similar to *Lb. rhamnosus*-loaded nanofibers here, whereas the other nanofibes were a little smaller, with the lowest mean diameter of 91 ± 19 nm for *Lb. plantarum*-loaded nanofibers. In line with previous studies, the addition of the bacteria to the PEO solutions did not only changed the viscosity, but also the dispersion conductivity. A prominent influence of conductivity over viscosity might lead to the decreased nanofiber diameters seen for the bacteria-loaded nanofibers [19,26].

While observing the bacteria-loaded nanofibers using scanning electron microscopy, we could clearly distinguish incorporated cells in nanofibers. Thus, electrospinning provided complete incorporation of the bacteria, even for the strain with the largest cells among those studied here (i.e., *Lb. delbrueckii* ssp. *bulgaricus*; cell length, 10.82 µm). As observed previously [26], although the morphology of the bacterial cells in the nanofibers was similar to their morphology obtained after air-drying the bacterial dispersions, some of the cells were flattened due to their drying and dehydration, or to the mechanical stress they had undergone [41].

All of these species of lactic acid bacteria that were incorporated survived the electrospinning; however, the theoretical and experimental bacterial loading differed. The electrospinning process results in a high incorporation efficiency of drugs [42] or bacteria [19] and here, the difference between both loadings was probably due to the decrease of bacterial viability. There were no correlations between the growth characteristics, viscosity of the dispersion, or zeta potential of the lactic acid bacteria and their survival. On the other hand, the decrease in viability of the bacteria in the nanofibers correlated with the hydrophobicities of the cells. Hydrophobicity depends on the surface structures of the bacterial cells, such as lipoteichoic acid, S-layer proteins, outer membrane proteins and lipids, surface fibrils, and various fimbriae or core oligosaccharides [43]. Considerable differences among the surface properties of lactic acid bacteria were also seen in our previous study [44]. We hypothesize that the hydrophobic surface offers better protection from the hydrophilic solution in which the bacteria are dispersed.

Another possible predictor of bacterial viability in these nanofibers is their extreme morphology. *Lb. delbrueckii* ssp. *bulgaricus* had by far the largest cells and the lowest viability, as the only species with a >2 log unit decrease in viability. This might be a consequence of the large bacteria surface per cell. The viability was not affected by the direction of electrospinning, which thus excluded gravitational effects.

To summarize, while the electrospinning process is feasible regardless of the species of lactic acid bacteria used, the prediction of viability is challenging and will require the testing of individual strains.

## 5. Conclusions

In the present study, we incorporated a range of safe lactic acid bacteria into PEO-based nanofibers, several of which are confirmed probiotics. All of the lactic acid bacteria remained viable after their incorporation into the nanofibers using an electrospinning procedure appropriate for PEO solutions, without the need for additional optimization. However, the survival of these lactic acid bacteria differed across two log units, and despite the determination of the physical and growth characteristics and the viscosities of the bacterial dispersions, no clear relationships were seen between the survival and the parameters measured. This suggests that the prediction of bacterial survival during the electrospinning process is challenging, and that the formulation of each particular bacterial strain will need to be optimized separately. The exceptions are the cell hydrophobicity and the extreme morphological characteristics of the bacteria (e.g., greatest size for *Lb. delbrueckii* ssp. *bulgaricus*), which offer some indications in terms of bacterial survival after their incorporation into nanofibers. The developed nanofiber-based delivery systems could be useful for the delivery of lactic acid bacteria to mucosal surfaces, such as oral, nasal, or vaginal surfaces.

## Figures and Tables

**Table 1:** Bacterial strains used in this study, and some of their properties.

Strain	Source	Fermentation Type	Genome Size [bp]	Reference
***Lactobacillus sp.***				
*Lb. acidophilus* ATCC 4356	Infant feces	Homofermentative	1956.699	[29]
*Lb. delbrueckii* ssp. *bulgaricus* ATCC 11842	Yoghurt	Homofermentative	1864.998	[30]
*Lb. casei* ATCC 393	Cheese	Facultative heterofermentative	2924.929	[31]
*Lb. gasseri* ATCC 33323	Human isolate	Homofermentative	1894.360	[32]
*Lb. paracasei* ATCC 25302	Milk product	Facultative heterofermentative	2991.737	NCBI
*Lb. plantarum* ATCC 8014	n/a	Facultative heterofermentative	3254.764	NCBI
*Lb. reuteri* ATCC 55730	Breast milk	Heterofermentative	2036.000	[33]
*Lb. rhamnosus* ATCC 53103	Human intestine	Homofermentative	3005.051	[34]
*Lb. salivarius* ATCC 11741	Infant feces	Homofermentative	1956.699	NCBI
***Lactococcus sp.***				
*Lactococcus lactis* ssp. *cremoris* MG1363	Cheese	Homofermentative	2529.478	[35]

n/a: not available, NCBI: National Center for Biotechnology Information, ATCC: American Type Culture Collection.

**Table 2:** Selected physical characteristics of the bacterial species used in this study.

Bacteria Species	Average Cell Width [µm]	Average Cell Length [µm]	Zeta Potential [mV]	Hydrophobicity [%]	Mass of 1 × 10^10^ Bacterial Cells [mg]
*Lb. acidophilus*	0.56 ± 0.04	1.28 ± 0.26	−9.1 ± 5.0	78.6 ± 1.0	0.65
*Lb. delbrueckii* ssp. *bulgaricus*	0.51 ± 0.07	10.82 ± 3.31	−9.4 ± 5.5	28.5 ± 3.9	9.14
*Lb. casei*	0.58 ± 0.06	1.54 ± 0.36	−0.4 ± 5.5	11.3 ± 0.5	4.22
*Lb. gasseri*	0.65 ± 0.07	4.68 ± 1.43	−7.9 ± 4.6	92.5 ± 2.1	5.59
*Lb. paracasei*	0.68 ± 0.09	2.05 ± 0.49	−23.9 ± 4.4	36.4 ± 4.8	2.13
*Lb. plantarum*	0.52 ± 0.04	1.33 ± 0.29	−12.7 ± 4.0	74.2 ± 2.3	0.54
*Lb. reuteri*	0.72 ± 0.08	1.43 ± 0.37	−13.7 ± 5.7	71.9 ± 5.8	1.04
*Lb. rhamnosus*	0.64 ± 0.08	2.16 ± 0.51	−3.9 ± 4.4	31.5 ± 7.7	16.31
*Lb. salivarius*	0.70 ± 0.07	1.39 ± 0.31	−11.7 ± 4.6	90.1 ± 1.3	1.92
*L. lactis*	0.53 ± 0.05	0.74 ± 0.18	−12.8 ± 5.5	24.2 ± 6.3	0.48

**Table 3:** Growth rates and lag times of the *Lactobacillus* spp. and *L. lactis* determined from their growth curves.

Bacterial Species	Growth Rate [/h]	Lag Time [h]
*Lb. acidophilus*	0.158 ± 0.005	2.31 ± 0.09
*Lb. bulgaricus*	0.066 ± 0.001	8.14 ± 0.15
*Lb. casei*	0.116 ± 0.002	3.41 ± 0.07
*Lb. gasseri*	0.118 ± 0.004	1.60 ± 0.15
*Lb. paracasei*	0.102 ± 0.010	5.50 ± 0.23
*Lb. plantarum*	0.199 ± 0.002	2.52 ± 0.21
*Lb. reuteri*	0.193 ± 0.009	1.82 ± 0.51
*Lb. rhamnosus*	0.125 ± 0.001	1.92 ± 0.34
*Lb. salivarius*	0.297 ± 0.028	1.11 ± 0.08
*L. lactis*	0.326 ± 0.013	0.84 ± 0.07

**Table 4:** Viability of the lactic acid bacteria in PEO solution before and after their incorporation into nanofibers.

Bacterial Species	Theoretical Bacteria Loading (log CFU/mg)	Experimental Bacteria Loading (log CFU/mg)	Decrease in Viability (log CFU/mg)
*Lb. acidophilus*	9.89 ± 0.18	9.18 ± 0.21	0.71
*Lb. bulgaricus*	8.74 ± 0.15	5.91 ± 0.15	2.83
*Lb. casei*	8.85 ± 0.21	7.33 ± 0.19	1.52
*Lb. gasseri*	9.30 ± 0.18	9.20 ± 0.11	0.09
*Lb. paracasei*	9.53 ± 0.12	8.02 ± 0.18	1.51
*Lb. plantarum*	9.99 ± 0.06	8.70 ± 0.38	1.29
*Lb. reuteri*	9.76 ± 0.13	9.32 ± 0.04	0.44
*Lb. rhamnosus*	8.55 ± 0.13	7.41 ± 0.16	1.14
*Lb. salivarius*	9.21 ± 0.06	6.75 ± 0.07	0.73
*L. lactis*	9.06 ± 0.14	8.19 ± 0.33	0.87
